# Interaction vesicles as emerging mediators of host‐pathogen molecular crosstalk and their implications for infection dynamics

**DOI:** 10.1002/1873-3468.70055

**Published:** 2025-05-01

**Authors:** Bruna Sabatke, Izadora Volpato Rossi, Marcel I. Ramirez

**Affiliations:** ^1^ Graduate Program in Microbiology, Pathology and Parasitology Federal University of Paraná Curitiba Brazil; ^2^ EVAHPI – Extracellular Vesicles and Host‐Parasite Interactions Research Group, Carlos Chagas Institute (Fiocruz‐PR) Curitiba Brazil; ^3^ Graduate Program in Cell and Molecular Biology Federal University of Paraná Curitiba Brazil

**Keywords:** extracellular vesicles, host‐pathogen communication, immune evasion, infection persistence, interaction vesicles

## Abstract

Extracellular vesicles (EVs) are critical in cell communication, transfer of biomolecules, and host‐pathogen interaction. A newly identified subset, “interaction vesicles” (iEVs), forms through host‐pathogen contact, merging membrane elements from both. These iEVs may arise through multiple mechanisms, including direct cell–cell contact, membrane contact sites, uptake and repackaging of foreign EVs, and post‐release fusion of EVs. These hybrid vesicles enable pathogens to modify host environments, aiding immune evasion and infection persistence. However, iEVs may also act in favor of the host, contributing to pathogen recognition and elimination. Advanced techniques, including proteomics and high‐resolution microscopy, are beginning to clarify their composition and fusion. Yet, isolating these hybrid EVs remains challenging. Overcoming these barriers could enhance understanding of infection mechanisms and support diagnostic and therapeutic innovation.

## Graphical abstract

Interaction extracellular vesicles (iEVs) are hybrid vesicles formed through host‐pathogen communication. They facilitate immune evasion, transfer pathogens' molecules, increase host cell uptake, and enhance virulence. This Perspective article illustrates the multifunctional roles of iEVs and highlights their emerging relevance in infection dynamics and host‐pathogen crosstalk.

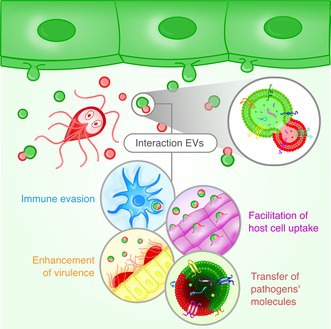

## Abbreviations

**AFM**, atomic force microscopy

**CRYO‐EM**, cryogenic electron microscopy

**CyTOF**, cytometry by time of flight

**DiI**, red fluorescent lipophilic dye (1,1′‐dioctadecyl‐3,3,3′,3′‐tetramethylindocarbocyanine perchlorate)

**DiO**, green fluorescent lipophilic dye (3,3′‐dioctadecyloxacarbocyanine perchlorate)

**EVs**, extracellular vesicles

**FRET**, Förster resonance energy transfer

**GFP**, green fluorescent protein

**HIV**, human immunodeficiency virus

**HSP70**, heat shock protein 70

**iEVs**, interaction vesicles

**LEVs**, large extracellular vesicles

**MHC**, major histocompatibility complex

**MS**, mass spectrometry

**Mtb**, *Mycobacterium tuberculosis*

**nFCM**, nano‐flow cytometry

**NTA**, nanoparticle tracking analysis

**OMVs**, outer membrane vesicles

**PAMPs**, pathogen‐associated molecular patterns

**PCR**, polymerase chain reaction

**PEG**, polyethylene glycol

**pH**, hydrogen potential

**PKH26**, red fluorescent dye for membrane labeling

**PKH76**, green fluorescent dye for membrane labeling

**RNA**, ribonucleic acid

**SEVs**, small extracellular vesicles

**SP‐ICP‐MS**, single particle inductively coupled plasma mass spectrometry

**VSV‐G**, vascular stomatitis virus protein

## Conceptual framework for understanding extracellular vesicles and interaction vesicles

Extracellular vesicles (EVs) are recognized as critical mediators of intercellular communication, responsible for transferring proteins, lipids, and nucleic acids between cells [1]. EVs include exosomes, microvesicles, and apoptotic bodies, each categorized based on distinct biogenesis pathways, sizes, and molecular cargos. In recent years, EVs have become a focal point in host‐pathogen interaction studies, shedding light on how pathogens can exploit host‐derived vesicles to facilitate infection, immune evasion, and pathogenic persistence [2].

**Fig. 1:**
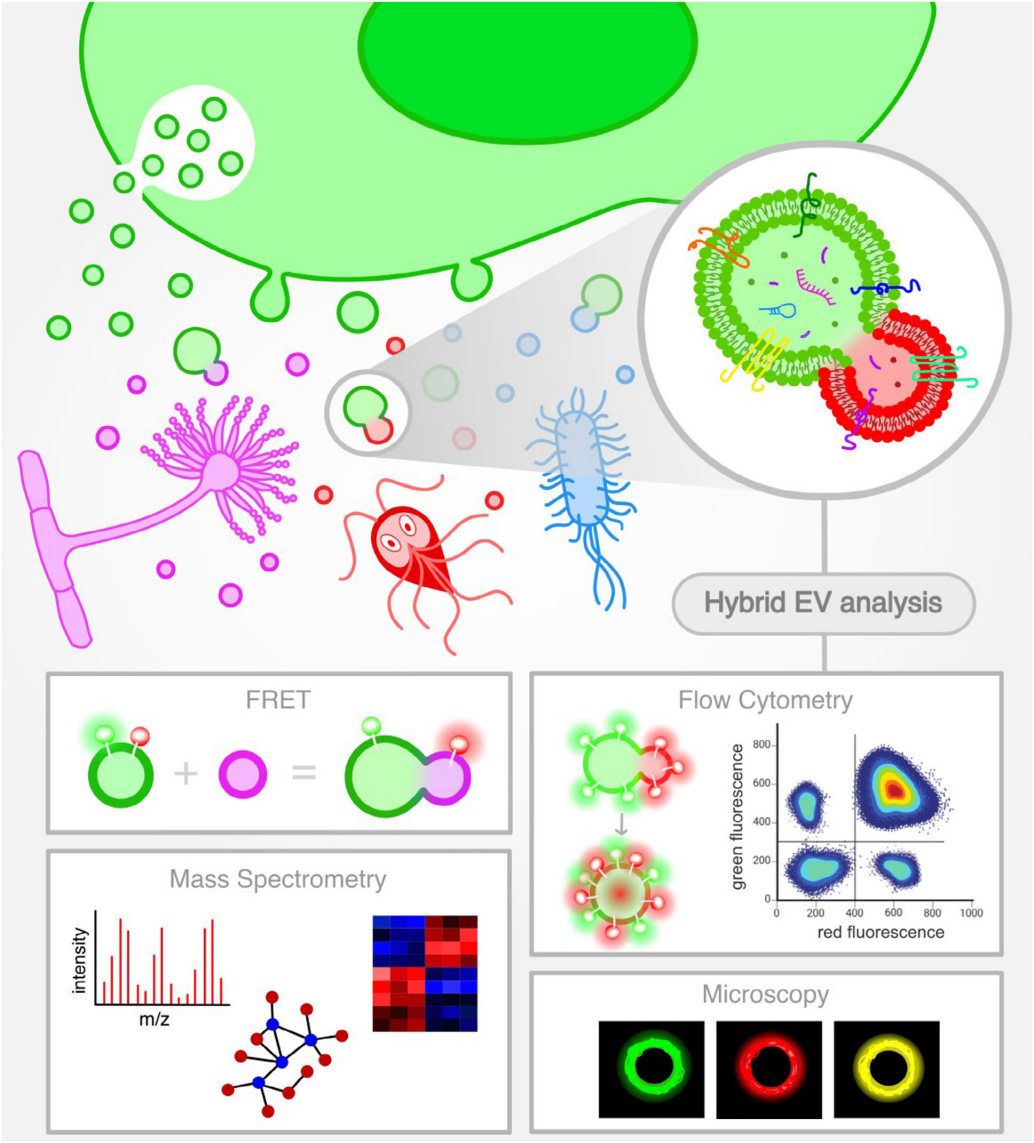
Biogenesis and analysis methods of interaction extracellular vesicles (iEVs). Interaction EVs contain a mixture of host‐ and pathogen‐derived components that may arise through multiple mechanisms, including direct cell–cell contact, membrane contact sites, uptake and repackaging of foreign EVs, and post‐release fusion of EVs. iEVs specifically refer to EVs containing both host‐ and pathogen‐derived components, distinguishing them from generic EV interactions. The most suitable strategies for showing iEV formation include Föster resonance energy transfer (FRET), dual‐labeled flow cytometry, and high‐resolution imaging techniques. While omics approaches play a crucial role in characterizing iEV composition, these methods primarily confirm molecular content rather than directly demonstrating vesicle fusion or formation. This figure primarily focuses on the post‐fusion generation of iEVs, which is the central theme of this perspective. Other potential mechanisms, such as direct uptake and membrane contact sites, are discussed in the text. Designed in inkscape (Open Source Scalable Vector Graphics Editor).

A particularly compelling concept that has emerged is that of “interaction vesicles” (iEVs) (Fig. 1). We propose defining interaction vesicles as extracellular vesicles that contain a mix of host and pathogen‐derived components, which may arise through multiple mechanisms, including direct cell–cell contact, membrane contact sites, uptake and repackaging of foreign EVs, and post‐release fusion of EVs. While direct cell contact may contribute to iEV formation in certain cases, it is not a strict requirement for their generation. These hybrid structures integrate membrane components from both entities, representing a unique form of molecular crosstalk that can profoundly affect infection dynamics and disease outcomes [3, 4, 5, 6]. Direct cell contact may facilitate iEV formation by enabling localized vesicle exchange at tight junctions, invaginations, or transient fusion events where membrane material is transferred between the host and pathogen. For example, some intracellular pathogens establish intimate contact with host membranes, which may promote the direct incorporation of host components into pathogen‐derived vesicles. The vesicles are characterized by their dual‐origin membranes, containing a mixture of host‐derived and pathogen‐derived proteins, lipids, and other molecular components. In our definition, an EV is classified as an iEV only if it contains both host and pathogen membrane components, distinguishing it from vesicles that solely contain luminal pathogen cargo. This unique composition enables iEVs to serve as specialized platforms for the exchange of molecular signals, facilitating immune evasion, enhancing pathogen virulence, and modifying the host cellular environment to favor persistent infection.

iEVs suggest a broader role for EVs beyond traditional communication: they embody a mechanism that enables pathogens to co‐opt host resources in ways that may reshape host‐pathogen dynamics and enhance virulence or immune modulation [7, 8]. While recent studies suggest that iEVs may play a role in immune modulation and pathogen adaptation, definitive evidence for their precise mechanisms and functions remains limited. This perspective presents an emerging concept based on initial observations and requires further experimental validation.

## Balanced perspective on the role of interaction vesicles

iEVs seem to serve dual roles within the host‐pathogen interface, functioning both in favor of the pathogen and the host. On the one hand, these vesicles can enhance immune activation and facilitate pathogen clearance by delivering pathogen‐associated molecular patterns (PAMPs) that trigger innate immune responses, stimulating cytokine production and antigen presentation [9, 10]. Additionally, they can act as delivery systems for immunogenic antigens, aiding in the development of adaptive immune responses and promoting pathogen elimination.

Conversely, pathogens exploit iEVs to evade immune surveillance and establish persistent infections. By incorporating host‐derived molecules into their membrane, these vesicles can mask pathogen antigens, avoiding recognition by the immune system. Moreover, they may deliver regulatory factors that suppress immune responses, such as pathogen‐derived immunomodulatory molecules, including surface lipophosphoglycans from *Leishmania* or cysteine proteases from *Giardia*, and facilitate pathogen dissemination by modulating cellular processes like adhesion, migration, and invasion [11, 12].

Diverse types of cargo within iEVs, including RNA, proteins, and lipids, contribute to their dual immune‐modulatory effects. These vesicles can enhance or suppress immune responses depending on their composition and interaction with host cells. Thus, the interplay between iEVs and the host immune system presents a complex landscape in which these vesicles may act as both weapons and shields. For example, iEVs may carry pathogen‐derived surface proteins that mimic host ligands, potentially leading to increased internalization via host cell receptors such as integrins and scavenger receptors. However, it is also plausible that host cells internalize iEVs primarily through their existing recognition pathways, with pathogen molecules being incidentally taken along rather than actively mimicking host ligands. While some studies suggest that pathogen‐derived molecules can interact with host receptors, direct evidence for widespread host‐ligand mimicry in iEVs remains limited and warrants further investigation [2, 11]. This enhanced uptake can result in the modulation of intracellular signaling cascades, promoting pathogen survival and immune evasion. While many EV‐mediated functions have been observed in host and pathogen‐derived vesicles separately, iEVs may integrate these roles by carrying both host and pathogen‐derived factors, thus creating a hybrid influence on immune modulation.

Furthermore, iEVs can alter cytokine signaling by delivering specific lipid mediators and proteins that suppress or activate immune responses. The presence of pathogen‐derived lipids such as lipophosphoglycan in *Leishmania*‐derived vesicles has been shown to downregulate pro‐inflammatory cytokine production, contributing to immune suppression and chronic infection [13]. Conversely, vesicles containing host‐derived immune‐modulatory proteins, such as HSP70 and MHC class I molecules, can trigger inappropriate immune activation, leading to immune evasion or exacerbated inflammation [14].

These hybrid vesicles may also modulate immune cell activation by delivering molecular cargo that interferes with antigen presentation and T‐cell activation. Examples include *Giardia intestinalis*‐derived vesicles carrying surface proteins that interfere with dendritic cell maturation, impairing the host's ability to mount an effective adaptive immune response [15]. Additionally, *Mycobacterium tuberculosis*‐derived vesicles containing lipoglycans have been shown to suppress macrophage activation, allowing the bacteria to evade immune clearance [16]. While *Giardia*‐ and *Mycobacterium*‐derived EVs have been implicated in immune modulation, only those containing a mixture of host‐ and pathogen‐derived components qualify as iEVs. In the case of *G. intestinalis*, the presence of host‐derived molecules in these vesicles remains to be confirmed.

iEVs thus represent an overlooked aspect of cross‐kingdom communication, bridging the gap between microbial and host cell interactions. Their ability to carry a diverse molecular cargo, including nucleic acids, lipids, and proteins, enables pathogens to manipulate host responses at multiple levels [1, 17]. In this point, RNA is a particularly significant component of iEVs, as it serves as a universal signaling molecule in cross‐kingdom communication. Small RNAs within pathogen‐derived EVs have been shown to modulate host immune responses by interfering with gene expression [18, 19, 20, 21].

This intricate interplay highlights the need for further research to elucidate the molecular determinants that govern the balance between pro‐host and pro‐pathogen roles, which will be crucial in developing targeted therapeutic interventions.

## Examples of interaction vesicles in pathogen‐host systems

The existence and function of iEVs have been documented across various pathogens, with growing evidence demonstrating their significance in infection processes. In *Trypanosoma cruzi*, studies have shown that iEVs carry a composite of both parasite and host cell membrane proteins, which can help the parasite evade host immune detection and facilitate its survival within host cells [11, 12]. Recent evidence by Rossi *et al*. [22] suggests that *T. cruzi*‐infected cells release large extracellular vesicles (LEVs) containing both host and parasite proteins. It remains uncertain whether individual vesicles carry components from both sources. Nonetheless, these vesicles play a vital role in manipulating host responses to benefit the parasite's intracellular persistence and, by extension, the chronicity of diseases such as Chagas disease.

Bacterial pathogens like *Mycobacterium tuberculosis* (Mtb) also produce hybrid vesicles, comprising components from both bacterial and macrophage membranes. These iEVs help modulate immune responses by reducing macrophage activation, thus fostering an environment conducive to bacterial survival. Regarding the formation of hybrid or iEVs, current evidence does not conclusively demonstrate that bacterial EVs are encapsulated within mammalian host EVs during their biogenesis or release. However, both bacterial and host cells release EVs that can interact within the extracellular environment, potentially influencing each other's functions and contributing to the complexity of host‐pathogen interactions. Studies have shown that macrophages infected with Mtb release two separate and largely distinct populations of extracellular vesicles. One population carries host exosomal markers such as CD9 and CD63, while the other is enriched with Mtb‐derived components like lipoglycans and lipoproteins. Although these vesicles are similar in size, they exhibit different densities, as demonstrated by their separation using sucrose gradient centrifugation [16]. Despite this evidence, the colocalization of *Mycobacterium* and macrophage antigens within each subpopulation suggests the existence of a variable percentage (4–17.5%) of hybrid vesicles that could play a significant role in modulating the immune system and regulating the infections.

Some studies have explored the strategy of artificially fusing EVs to unite components into a single structure. For example, bacterial outer membrane vesicles (OMVs) from *Escherichia coli* and attenuated *Salmonella* have been artificially fused with tumor cell membranes (B16‐F10 melanoma) and erythrocyte membranes to create hybrid vesicles. These studies demonstrated applications of these fused EVs in targeted immunotherapy, enhanced anticancer vaccination, and antibacterial treatments by combining immunogenicity with immune evasion properties, leveraging combined functionalities of both membrane types [9, 10].

Additionally, proteomic analyses of EVs in *Candida albicans* THP1‐derived EVs, both from control and from *Candida*‐infected macrophages [23], have shown the presence of both fungal and host molecules, suggesting that fusion between fungal and host cell membranes may occur. This fusion enables the EVs to carry a mixed cargo of fungal and host cell proteins, lipids, and other molecules, providing a mechanism for fungi to deliver virulence factors directly into host cells, influencing host‐pathogen interactions and immune modulation. Although proteomic studies have not demonstrated fusion, components from both pathogens and hosts have been identified, increasing the likelihood of iEVs. However, the hypothesis that these may be separate vesicles originating from each source cannot be ruled out.

The complexity of viral EVs warrants particular attention. EVs from virus‐infected cells carry both host and viral components, including immunosuppressive molecules that aid in immune evasion. Unlike other interaction vesicles, viral EVs do not necessarily require direct fusion of membranes between host and pathogen, as viruses inherently integrate into host membranes during replication. This distinction places viral EVs in a unique category within the broader landscape of extracellular vesicles containing mixed host and pathogen‐derived materials. Virion‐containing vesicles further illustrate how these hybrid vesicles are tailored for long‐term immune evasion and sustained infections. EVs from virus‐infected cells carry both host and viral components, including immunosuppressive molecules that aid in immune evasion. Unlike other iEVs, viral EVs do not necessarily require direct fusion of membranes between host and pathogen, as viruses inherently integrate into host membranes during replication. This distinction places viral EVs in a unique category within the broader landscape of extracellular vesicles containing mixed host and pathogen‐derived materials. EVs from virus‐infected cells carry both host and viral components, including immunosuppressive molecules that aid in immune evasion.

A key distinction must be made between EVs released from infected cells and virion‐containing vesicles. EVs from infected cells are host‐derived vesicles carrying altered cargo due to the infection, whereas they rather than existing as a separate subclass. EVs carrying viral material represent a distinct form of interaction vesicle, as their composition inherently results from the close integration of viruses with host cellular machinery. These vesicles can package viral proteins, RNA, and lipids alongside host‐derived components, influencing viral dissemination and immune evasion. Understanding this distinction is crucial, as viral pathogens, such as HIV, do not independently generate EVs but rather repurpose host EV machinery to facilitate viral dissemination and immune evasion. By hijacking the host's vesicular pathways, HIV can package viral components within EVs, enabling stealthy transport and evasion of immune detection [24, 25].

Together, these examples (Table 1) underscore how diverse pathogens manipulate EV structures to aid infection, bolster persistence, and facilitate immune evasion, underscoring the critical role of interaction vesicles in disease.

**Table 1:** Interaction EVs in different models of host‐pathogen interaction.

Organism	EV function	Methodology	Reference
*Trypanosoma cruzi*	Immune evasion and intracellular persistence	Expression of lysosomal‐associated membrane protein‐1‐GFP, fluorescence resonance energy transfer (FRET) assays, and proteomic analysis	[11, 12, 22]
*Mycobacterium tuberculosis*	Modulate immune responses	Exosomal markers (CD9, CD63) and sucrose gradient density separation	[16]
HIV	Immune evasion	Microscopy, proteomic and lipidomic analyses	[24]
*Candida albicans*	Carry virulence factors	Proteomic analyses	[23]

## Methods for studying vesicle fusion and techniques for characterizing hybrid vesicles

Fusion of biological membranes is common in biology, since membrane phospholipids are capable of reorganizing themselves, and proteins have fluid characteristics in membranes. Therefore, EV fusion would occur through mechanisms already described, involving proteins that would facilitate membrane junction. However, the fusion of EVs derived from pathogens with host cell membranes remains largely unexplored (Box 1).

Box 1 **Fusion between extracellular vesicles: how and why?**

Fusion between lipid membranes is known in many areas: in virology, it is the principle of virus infection; in pharmacology, it is used in the formulation of emulsions and liposomal solutions (synthetic phospholipid vesicles); in cell biology, the fusion of organelles such as lysosomes during endocytosis or the release of intracellular vesicles during exocytosis is mediated by membrane fusion. In the world of extracellular vesicles, mechanisms similar to those already known may govern the fusion between these nanoparticles, generating a single vesicle with shared components.

The sequence begins with interactions between the lipo‐phosphoprotein membranes of EVs and their cellular receptors. This binding can quickly lead to membrane fusion as lipid structures reorganize. Membrane proteins rearrange, and their hydrophobic sequences insert into the membrane of the interacting extracellular vesicle (iEV), promoting vesicle fusion. Initial EV interactions with other EVs likely resemble those observed between EVs and target cells, requiring high‐affinity binding of at least two surface proteins, such as syncytin‐1 and syncytin‐2, which bind to MFSD2a and are involved in EV‐cell fusion, particularly in the context of trophoblast EVs [26]. Other molecules like tetraspanins, annexins, and SNAREs may also participate. Further studies using techniques like mass spectrometry, co‐immunoprecipitation, and live‐cell imaging are needed to identify additional fusion‐related proteins and confirm their roles in EV‐mediated communication and fusion.

The interaction of pathogen‐derived vesicles with host cell membranes remains largely unexplored. Given evidence that vesicles from bacteria, fungi, and protozoa may directly interact with host membranes, understanding how molecules like SNAREs [27] or similar pathogen‐derived components contribute to fusion events could offer key insights into host‐pathogen interactions understanding and potential therapeutic strategies [25, 28].

The artificial induction of EV fusion with each other or lipid particles has been explored using methods like pH mediation, freeze–thaw, extrusion, PEG induction, and natural incubation [25]. These controlled experiments provide insights into the physicochemical conditions—such as membrane composition, charge, and fluidity—that may also regulate natural EV‐EV fusion in biological environments [25]. Mastering EV fusion with liposomes, EVs, and cells has potential for translational applications, as their high load capacity, biocompatibility, and stability make them ideal for drug delivery and immunization.

Artificial membrane fusion strategies, such as pH‐mediated fusion, PEG induction, and extrusion, have been employed to study EV interactions and assess their potential roles in cargo delivery, immune modulation, and intercellular communication. These approaches provide insights into how natural fusion events may contribute to host‐pathogen interactions.

Takeda *et al*. [29] demonstrated that small extracellular vesicles (sEVs) derived from mammalian cells and bacterial outer membrane vesicles fuse with endosomes more efficiently than artificial liposomes, highlighting the role of vesicle composition in fusion dynamics. Other studies, such as the use of R18‐labeled EVs with the plasma membrane of bone marrow‐derived dendritic cells [30], provide a speculative extension of this fusion model, although definitive evidence remains limited. These findings provide a foundation for understanding fusion events between extracellular vesicles from pathogens and host cells.

The characterization of fused EVs employs a strategy of combining conventional EVs characterization techniques with membrane fusion characterization methods to validate the fusogenic event. Imaging techniques, such as confocal microscopy and electron microscopy, allow the visualization of EVs. Using optimal and unique markers for each vesicle subpopulation, it is possible to observe the dynamics of fusion and vesicle formation during host‐pathogen interactions [31]. Confocal microscopy is particularly useful because it allows real‐time monitoring of fusion events. Using a pair of markers, such as PKH26 (red fluorescence) and PKH67 (green fluorescence), individually labeling pathogen EVs and host cell EVs, it is possible to identify hybrid EVs through the emission of yellow fluorescence, similar to that developed in other models [32, 33, 34]. However, it is essential to note that these approaches, while promising, often rely on engineered systems and therefore should be framed as speculative extensions rather than definitive evidence.

Super‐resolution microscopy goes further, providing a detailed view of vesicle structure at the molecular level, which is particularly useful for examining membrane fusion and cargo exchange at high resolution [35]. Also, emerging techniques like cryo‐electron microscopy (cryo‐EM) provide three‐dimensional structural data, enhancing our ability to visualize membrane‐bound structures in detail, including unique aspects of hybrid vesicles that are not detectable with traditional methods [1, 36].

Using independent fluorophores for each subpopulation or through immunophenotyping of EVs with unique markers of EVs from pathogens and hosts, it is possible to study fusion events using flow cytometry. Flow cytometers can indicate those vesicles found with double labeling [37, 38]. Furthermore, the possibility of performing EV sorting opens the way for studies of hybrid double‐positive EVs by other methods. These approaches, while innovative, also highlight the challenges of interpreting data from engineered systems versus naturally derived EVs, requiring careful delineation in the literature.

Förster resonance energy transfer (FRET) is a powerful technique used to study molecular interactions and distances at the nanoscale [39]. FRET can be used to evaluate EVs fusion, in which EVs from one origin labeled with the fluorophore pair interact with unlabeled EVs from another origin. By measuring the change in fluorescence emission intensity of the donor and acceptor fluorophore, the fusion phenomenon between EVs can be evaluated [34, 40]. FRET techniques can confirm true vesicle fusion by ensuring fluorochromes are localized intraluminally rather than on the surface, as surface‐bound vesicles may produce misleading signals due to clustering. Encapsulating donor and acceptor fluorophores, such as DiI/DiO or mCherry/GFP, allows FRET detection specifically when cargo mixing occurs within a single vesicle. To exclude surface binding artifacts, quenching agents like trypan blue or trypan red can eliminate external signals, and protease treatments can degrade surface‐bound proteins.

To dissect the molecular composition of hybrid vesicles, proteomic and lipidomic analyses are essential. Mass spectrometry (MS) enables researchers to identify specific proteins and lipids from both host and pathogen within a single vesicle [17]. These methods equip researchers to explore the distinctive properties of interaction vesicles, advancing the field's understanding of their roles in infection. Advanced MS techniques, such as single‐particle inductively coupled plasma mass spectrometry (SP‐ICP‐MS) and mass cytometry (CyTOF), enable high‐resolution characterization of EVs at the single‐vesicle level. These methods offer high sensitivity and multiplexing capabilities, detecting low‐abundance molecules and distinguishing between different EV subpopulations. However, challenges remain, including sample purity, instrumental limitations, and complex data interpretation. Despite these hurdles, MS‐based single‐vesicle analysis continues to evolve, improving our understanding of EV heterogeneity and their functional roles in biological systems [41].

Biological analyses commonly used in cell biology and biochemistry laboratories, such as western blots and polymerase chain reaction (PCR), can help us understand the function and composition of EVs. However, to date, these techniques can only be used on a set of isolated EVs and are therefore insufficient to identify the individual biophysical and biochemical characteristics of each EV. Thus, new technologies aim to analyze single particles rather than sets and can be used to characterize hybrid EVs, such as Nanoparticle Tracking Analysis (NTA), Nanoflow Cytometry (nFCM), and Atomic Force Microscopy (AFM) [42].

Optical tweezers can aid in the study of hybrid EVs, as they allow precise and non‐invasive manipulation of these EVs, providing detailed information about their physical and functional properties. With this approach, it is possible to measure intervesicular forces, membrane elasticity, and molecular interactions with proteins or other biomolecules, contributing to the understanding of processes such as cellular recognition, content delivery, and fusion mechanisms [43, 44, 45].

Some progress has already been made towards evaluating the process of delivery of cargo from EVs to recipient cells, which could also be used to study nanoparticle fusion. Bioluminescence reporter genes have demonstrated the functional delivery of proteins from one vesicle to another, such as through Cre‐LoxP [46] and split luciferase [47, 48]. While these methods provide insights, they often involve experimental systems and should not be mistaken for natural EV fusion events. Some authors have engineered EVs to contain fusion proteins on their surface (as viral fusogens, i.e., vascular stomatitis virus protein (VSV‐G)), which increases the effectiveness of the fusion process [25, 36, 38, 48]. Furthermore, the generation of fused EVs from distinct origins can be facilitated by other methods, such as extrusion, pH alteration, freeze–thaw cycles, addition of polyethylene glycol (PEG), and natural incubation [32, 33, 34, 49]. This strategy has not been employed with EVs from pathogens yet and remains a speculative avenue for future exploration.

The study of iEVs relies on advanced methods capable of distinguishing vesicle origins, fusion events, and membrane compositions. A key challenge in the study of EVs is distinguishing between vesicles that are merely attached to target cells and those that have truly fused. Confocal and super‐resolution microscopy allows real‐time visualization of these interactions, while flow cytometry detects fusion events using dual‐fluorophore labeling. Biochemical assays, such as proteinase K treatment, remove surface‐bound proteins, isolating internalized markers. FRET further confirms membrane fusion by measuring energy transfer between fluorophores. These techniques are essential for accurately studying EV interactions, offering insights into their role in infection and therapeutic potential.

## Technological challenges in identifying and analyzing interaction vesicles

Despite technological advancements, challenges remain in identifying and analyzing iEVs. A key issue is distinguishing vesicles from host or pathogen sources from hybrids. Traditional techniques often lack the sensitivity to detect subtle compositional differences, such as membrane protein and lipid variations [50], complicating the accurate characterization of these vesicles and the identification of pathogen‐specific markers in host membranes.

Current EV purification methods are often insufficient for isolating iEVs, as they co‐sediment with similar‐sized vesicles. Promising approaches like single‐vesicle analysis allow the study of individual vesicles, potentially revealing unique signatures of hybrid structures [51]. Combining high‐resolution molecular imaging with proteomic and lipidomic techniques could improve vesicle identification, enhancing our understanding of pathogen‐host interactions. Further research is needed to explore the role of iEVs in disease mechanisms and inform new diagnostic and therapeutic strategies. The molecular signals governing fusion between pathogen and host membranes remain unknown, and it is unclear whether this phenomenon serves as a host defense or a pathogen virulence strategy.

## Relevance, open questions, and challenges of interaction vesicles

iEVs represent a novel mechanism of bidirectional cargo exchange between pathogens and host cells, distinct from the independent uptake of parasite‐ and host‐derived EVs. By merging molecular components from both sources, these vesicles may alter recognition, uptake efficiency, and functional impact on infection dynamics. Their ability to selectively transfer immune‐modulatory molecules, adhesion receptors, and stress‐response proteins suggests a role in immune evasion, enhanced virulence, and pathogen persistence. For instance, if a fungal EV fuses with a host‐derived EV, the presence of host receptors might improve uptake, while parasite–host hybrid EVs could shield antigens, preventing immune clearance. Key molecules such as integrins and lectins may govern these processes, facilitating selective vesicle fusion and enhancing pathogen adaptation.

Despite their potential significance, iEVs remain an overlooked aspect of cross‐kingdom communication. They possibly influence microbial interactions, horizontal gene transfer, and host immune responses. Fundamental questions arise: What determines vesicle fusion specificity? Are there biophysical constraints, such as lipid composition or membrane charge, limiting their formation? Understanding these mechanisms could reveal novel therapeutic targets, such as blocking vesicle hybridization to disrupt pathogen communication. Exploring iEVs not only challenges conventional views of infection but also opens new avenues for controlling host–pathogen interactions.

Efforts should be made to better understand the impact of iEVs in natural biological systems and disease pathologies to accurately assess their role in infection dynamics. This includes conducting *in vivo* studies to validate their formation and function, characterizing their molecular composition in different physiological contexts, and elucidating their mechanisms of action in host‐pathogen interactions. Advancing these areas of research will be essential for determining their potential as therapeutic targets for disease intervention.

## Author contributions

BS, IVR and MR contributed to conceptualization, original draft preparation and review and editing. MR contributed to funding acquisition and supervision. All authors have read and agreed to the published version of the manuscript.
